# Behavioral and Developmental Changes in Rats with Prenatal Exposure of Mirtazapine

**DOI:** 10.3797/scipharm.1004-02

**Published:** 2010-06-30

**Authors:** Jasmita Sahoo, Ashok K. Pattnaik, Nibha Mishra

**Affiliations:** Department of Pharmaceutical Sciences, Birla Institute of Technology, Mesra, Ranchi 835215 India

**Keywords:** Prenatal exposure, Behavioral development, Mirtazapine

## Abstract

Mirtazapine is an often used antidepressant drug; however insufficient information is available regarding its safety during pregnancy. Therefore, this work was initiated to study the effect of prenatal exposure of mirtazapine on postnatal developments of rats. The study was conducted on pregnant rats to observe the safety profile of mirtazapine in comparison to control. The percentage weight gain, gestation period and litter size of the rats treated with double therapeutic dose (DTD) was significantly lower than the rats treated with therapeutic dose (TD) and rats of control group. However the litter size of the TD treated rats was also found smaller than the control. The offspring were examined through battery of test in order to evaluate their developmental neurotoxicity. The test includes the assessment of postnatal growth, reflex ontogeny, neuromotor abilities, activity level, emotional reactivity and learning ability. The DTD exposure negatively affected on overall growth of pups in comparison to TD exposed pups and control group. Further, the amine concentration in brain was also found significantly lower in DTD exposed pups. Therefore, this study reveals that the treatment of pregnant rats with TD and DTD decreases their litter size. In addition the prenatal exposure of DTD of mirtazapine negatively affects on neurodevelopment of rats.

## Introduction

Recent studies [1–3] suggest that the adverse fetal effects are associated with antidepressant drug exposure during pregnancy. However, further studies are necessary to measure the consequences of newer antidepressants fetal exposure such as mirtazapine. Mirtazapine is an atypical antidepressant agent, it is used not only for treatment of depression and anxiety but also for treatment of nausea and hyper emesis gravid arum [4, 5].

There is little information available on the safety of mirtazapine usage during pregnancy. In some studies [6–9] on mirtazapine exposure in pregnancy reported that out of 104 pregnancy outcomes; 77 live births, 1 stillbirth, 20 spontaneous abortions, 6 therapeutic abortions, and 2 major malformations were observed. In another fertility study [10] on rats, 100 mg/kg doses of mirtazapine were not affected on mating and conceptions, but it leads to pre-implantation losses. Further, the estrous cycle was found disrupted with 3 times or higher doses of maximum recommended human dose (∼5 mg/kg). These studies provided necessary indication and raised significant concern about the safe usage of mirtazapine during pregnancy. Therefore, the current investigation was undertaken to quantify the risk associated with the mirtazapine usage in pregnancy. Primarily, the physical and behavioral development were explore related to the prenatal exposure of mirtazapine.

## Results and Discussion

The *in utero* exposure of mirtazapine on postnatal development of offspring was explored and the two different doses i.e. double therapeutic dose (DTD) and (TD) were administered during 6^th^ to 20^th^ day of pregnancy. The pregnant rats were monitored for changes during gestation period and litter size. Further, the offspring were examined for neurobehavioral developments.

The physical development of offspring was assessed by observing for eye opening, pinna detachment and eruption of incisors. The behavioral ontogeny of pups was inspected with surface righting reflex test, negative geotaxis test and ascending wire mesh test. The motor development of pups was evaluated using swimming development test and rota rod test. The spatial memory of the pups was tested with Morris water maze test. Finally the pups were sacrificed and the brain amines were estimated.

### Maternal observations

**Fig. 1.**
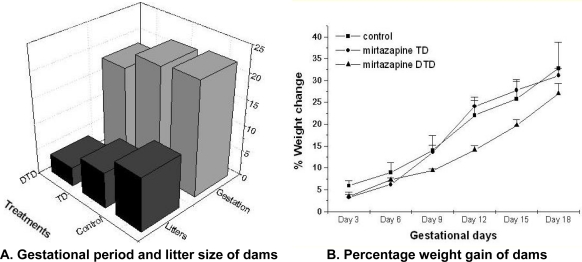
Effect of mirtazapine on pregnant rats. A: DTD treated dams showed significantly lower gestational period (p<0.05) and litter size (p<0.001) as compared to TD treated dams and control group dams. Litter size of TD treated dams was also significantly lesser (p<0.01) than control. B: Percentage weight gain of DTD treated dams was significantly lower (p<0.01) on gestational day 9, 12, 15 and 18 as compared to TD treated dams and control group dams. Data analysis: one way ANNOVA followed by post hoc tukey.

The pregnant rats were treated with TD or DTD during 6^th^ to 20^th^ day of pregnancy. The rats which were treated with DTD have shown significantly lower percentage weight gain on day 9 (F _2, 46_ = 7.65, p<0.01); on day 12 (F _2, 46_ = 8.58, p<0.01); on day 15 (F _2, 46_ = 9.22, p<0.01) and on day 18 (F _2, 46_ = 8.77, p<0.01) than TD treated rats and control groups rats (Fig.1.B). The gestational period of the DTD treated rats was significantly shorter (F_2, 21_ = 3.32, p<0.05) than TD treated and control group (Fig.1.A). One of the most remarkable observation was that, litter size of mirtazapine [DTD (F_2, 21_ = 18.73, p<0.001) or TD (F _1, 14_ = 13.42, p<0.01)] treated rats was significantly smaller than that of control (Fig.1.A).

The above results suggest that the mirtazapine possess feto-toxic effects. These observations are in congruence with earlier studies [6–9], which reports the spontaneous abortions and major malformation associated with mirtazapine exposure.

### Physical development

**Fig. 2.**
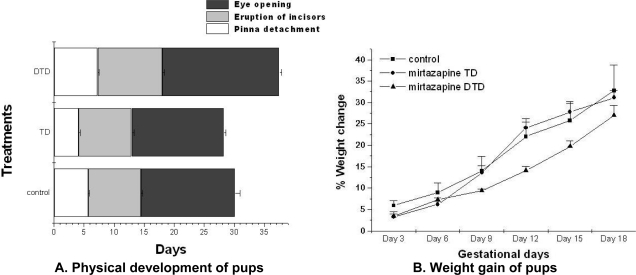
Effect of mirtazapine on physical developments. A: DTD exposed pups pinna detachment, eruption of incisors and pinna detachment was significantly (p<0.05) slower than TD exposed pups and control. B: DTD exposed litters gained significantly (p<0.05) lower weight on PND 0 and PND 3 than TD exposed litters and control group litters. Data analysis: one way ANNOVA followed by post hoc tukey.

The physical development of the DTD exposed pups were different from TD exposed and control group pups. DTD induced significant reductions in body weight on day 0 (F_2,46_ = 1.68, p<0.05) and day 3 (F_2,46_=1.33, p<0.05) than TD exposed and control pups (Fig 2, B).

The DTD exposed pups pinna detachment (F_2,46_ = 1.56, p<0.05), eruption of incisors (F_2,46_ = 2.11, p<0.05) and eyes opening (F_2,46_ = 2.48, p<0.05) was delayed in comparison to TD exposed pups and control (Fig 2, A).

### Behavioral development

The effect of *in utero* exposure of mirtazapine (TD or DTD) on behavioral development of pups was explored at various stages of life. The pups were tested for surface righting reflex activity from 4^th^ to 8^th^ PND. The drug exposed pups were found less active than the control pups: DTD exposed (F _2, 46_ = 8.55, p<0.01), TD exposed (F _2, 32_ = 5.62, p<0.01). The negative geotaxis movement of pups was tested from 8^th^ to 12^th^ PND. The DTD exposed pups took more time to turn to 180˚, on PND 8 (F_2, 46_ = 9.21; P<0.01), PND 10 (F_2, 46_ =8.67; P<0.01) and PND 12 (F_2, 46_ = 8.43; P<0.01)]) than TD exposed and control pups.

The pups were examined for ascending wire mesh activity on PND 14^th^ to 18^th^. The DTD exposed pups took more time than the TD exposed and control group pups specifically on PND 14 (F_2, 46_ = 7.46; P<0.01) and PND 18 (F_2, 46_ = 8.33; P<0.01)].

The swimming ability of pups was tested from 6^th^ to 12^th^ PND. The drug exposed pups were inefficient than the control pups: DTD (F_2, 46_ = 9.21; P<0.01) and TD (F_2, 32_ = 3.52; P<0.05). The pups’ motor ability was investigated on 22^nd^ and 59^th^ PND using rota rod. The DTD exposed pups spent shorter time on rod than the TD exposed and control pups: on PND 22 (F_2, 46_ = 2.13; P<0.05) and on PND 59 (F_2, 46_ = 1.69; P<0.05).

Thus, the above result reveals the prenatal exposure of mirtazapine affected the motor development of pups. The present study supports the earlier report [14, 15], which suggest that the children exposed with serotonin reuptake inhibitors, displayed subtle changes in motor development and in motor movement control. In addition, the various studies [16, 17] suggest that the prenatal exposure of most of the antidepressants affects on motor development of offspring.

### Emotional reactivity

**Fig. 4.**
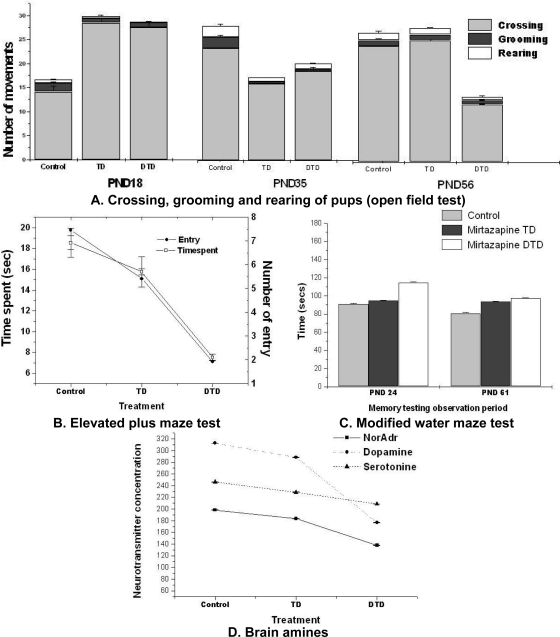
Effect of mirtazapine on behavior. A: Open field test: TD and DTD exposed pups were significantly (p<0.05) more active on PND 18 but less active on PND 56 compared to control. B: Plus maze test: DTD exposed pups spent significantly (p<0.01) less time and number of entries than TD exposed pups and control. C: Modified water maze: DTD exposed pups on PND 24 took significantly (p<0.05) more time than TD exposed pups and control. D: DTD exposed pup’s Noradrenaline & Dopamine was significantly (p<0.05) lesser, serotonin was similar to TD exposed pups and control. Data analysis: one way ANNOVA followed by post hoc tukey.

The exploratory activity of the pups was investigated on 18^th^, 35^th^ and 56^th^ PND. The pups which were *in utero* exposed to mirtazapine (both TD and DTD) were more active on PND 18 *i.e.* increase in total crossing (F_2, 46_ = 1.21; P<0.05). The pups which were *in utero* exposed to DTD were less active on PND 35 and 56 i.e. decrease in total crossing (F_2, 46_ = 1.78.; P<0.05) than control (Fig 4. A). The above discussed results were not conclusive.

The anxiety associated behavior of pups was explored on PND 30 using plus maze. The pups which were exposed to mirtazapine specifically DTD, were more anxious than TD exposed pups and control group pups. They executed lower number of entries (F_2, 46_ = 10.49; P<0.01); and spent lesser time (F_2, 46_ = 11.72; P<0.01) on open arm (Fig 4. B).

From the above study the DTD exposed pups were observed as more anxious than the TD exposed and control pups. The discussed anxious behavior may be due to the effect of mirtazapine on serotonin neurotransmission. The earlier reports [18–21] also reveal the increased anxiety and depression like behavior through serotonin transporter knockout mice. The additional studies [22–24] related to prenatal exposure of selective serotonin reuptake inhibitors followed to irritability and aggression in offspring.

### Cognitive response

The detrimental effects are potentially associated with exposure of substances throughout or later in pregnancy on neurobehavioral and cognitive development [25]. Therefore, effect of mirtazapine exposure on cognition and memory of the pups was studied on PND 24 and 61. The DTD exposed pups showed poor learning ability on PND 24 [they took more time (F_2, 46_ = 1.78; P<0.05) to escape to the platform in Morris water maze test] than the TD exposed and control group pups (Fig 4. C). Whereas on PND 61 the drug exposed pups were not different from control pups in their learning ability.

The present investigation did not provide any significant difference between cognition of TD exposed pups and control. However, the significant difference was observed between the DTD exposed pups and control pup on PND 24. The observe difference was may be due to any developmental neurotoxicity with higher doses. Further investigation may warrant consolidating the claim. The overall results are comparable with previous studies [26], where antidepressants produced in-significant effect of on cognition. Though, the identified studies varied considerably in their own methodology, including the age of the children studied, the scales and assessments used.

### Biochemical aspects

The amniotic fluid of drug treated rats was analyzed. The TD treated rat’s placental drug concentration was 8 ± 0.19 μg/ml (n=8) and the DTD rat’s placental concentration was 13 ± 0.15 μg/ml (n=8). Therefore, the concentration of drug in placenta increased with increase in drug dose. The concentration of amines of pup’s brain was estimated on PND 62. The Noradrenaline and Dopamine concentration was found significantly (F_2, 46_ = 4.69; P<0.05) lower in brains of DTD exposed pups in comparison to TD exposed and control (Fig. 4. D).

Therefore, the lower concentration of amines in the brain of DTD exposed pups may be responsible for their behavioral changes. The previous reports also suggest the tropic role of monoamines on brain morphogenesis [27–29].

## Experimental

### Animals

Thirty-six inbred Wistar albino rats, twenty-seven female and nine male (90–120 days old, weighing 120–150 gm) were selected. One male and three females were kept together for mating. Twenty four rats were confirmed pregnant by observing sperm in their vaginal smear and gestational day zero was assigned. All animals were maintained in the standard laboratory condition (25±2°C, humidity 55–60%) with alternate light and dark cycle (12/12 h). They were allowed standard pellet diet and water ad libitium. All experiments were performed as per Institutional Animals Ethics committee (IAEC), granting certification BIT/PH/IAEC/07/2007.

### Drug Administration

Mirtazapine was procured from Sun Pharmaceutical Industries, Jammu, India. Two doses were selected for study 3.6 mg/kg, therapeutic dose (TD) and 7.2 mg/kg, double the therapeutic dose (DTD) (http://www.genepharm.com.au). Twenty-four pregnant rats were divided as follows: control group (no treatment), TD group and DTD group. Drug was administered orally dissolved in distilled water, at 10 am daily (6th to 20th day of gestation period). Dose was decided regularly according to the weights of dams.

### Examination of dams and pups

Pregnant rat’s (dams) gestation period and percentage weight gain was recorded on every 3rd day. After delivery their litter size and litter weights were recorded. After birth, pups were allowed for 21 days weaning. During weaning period pups of each litter were weighed regularly on every 3rd day. Pups day of eye opening, pinna detachment and eruption of incisors were observed.

### Motor development and behavioral development studies

Behavior of pups was studied as follows: two pups of each mother rat, i.e. sixteen pups from each group were selected and they were subjected to the following behavioral tests: surface righting reflex, swimming development, negative geotaxis, ascending wire mesh, rotarod test, elevated plus maze, open field exploration, modified water maze (refer supporting information).

### Experimental protocol for motor development and behavioral development studies

#### Surface righting reflex

Each pup was placed in a supine position and allowed a maximum time of 2s to upright itself, two trials were given per day from 4th to 8th PND (post natal day). Day of achievement of righting reflex was recorded.

#### Swimming development

Pups were placed in water filled Plexi-glass container (40 x 30 x 14 cms) for 15 s on their PND 6, 8, 10 and 12. They were scored for angle of body (position of head in relation to surface of water) and limb movement.

#### Negative geotaxis

Pups were placed on an inclined (30°) surface with their heads facing downward. The time necessary to turn to 90° and subsequently to 180° was recorded. They were tested for 90s on PND 8 to PND 12.

#### Ascending wire mesh

The wire mesh (50 x 30 cms) was dipped in water bath at 26°C such that it is 31 cm above the water surface. The pups on PND 14 to PND 18 pups were placed with their quarter hind and tail dipping in the water. Day of achievement of the pups to the top within 30 s was recorded.

#### Rotarod test

The pups were given a preliminary test for 20 s then they were placed on the rod and turned at 25 rpm. Time taken by rat to stay on the rod without falling was recorded. Four such trials were done on PND 22 and 59.

#### Elevated plus maze

This task measures the response to fear of falling; it was performed by pups on PND 30. The maze consisted of 2 walled arms and 2 open arms that were elevated 38 cm above the floor. Number of entries and time spent on the open arm was recorded within 5 min of observation.

#### Open field exploration

A semi-soundproof room with a constant temperature was used. The home cage of animals was transferred into the room 1 h before testing. The floor was divided into 16 squares with plastic board (45 cm×45 cm). The rat was placed in the central area and latency to leave this area was recorded. Motor activity was expressed as a total number of squares crossed (with all four feet on one square) during the period of testing, horizontal activity (grooming) and vertical activity (total number of rearing). This study was performed on PND 18, 35 and 56

#### Modified water maze

The maze measured 100 cm in width and 80 cm in length was partitioned with wooden slants (12 cm width). On PND 24 and 61, the rats were placed in the one corner of the water maze (26°C). The time taken to escape from the maze to the platform was recorded. Each rat was given 6 consecutive trials with 1 min interval.

### Biochemical analysis

Brain of each rat was rapidly removed by decapitation on PND 62 and stored at −20^°^C till homogenization. The concentrations of Dopamine (DA), Noradrenaline (NA) and Serotonin (5-HT) were determined by the method of Ansell and Besin [11].

Mirtazapine was assayed as described by Santana and Bonato [12]. Absorption maximum was found at 315.4nm. The concentration of mirtazapine in the amniotic fluid of rats was determined using the method of Buznikov [13]. Two groups of pregnant rats were selected. One group was treated (6^th^ to 15^th^ day of gestation) with TD and other with DTD. On 15th day, 2hr after last dose, rats of both groups were sacrificed, uteri were dissected and the amniotic fluids were collected. The collected amniotic fluid was used for determining the concentration of mirtazapine.

### Statistical Analysis

Two pups per litter (one male and one female) altogether sixteen pups per group were randomly selected. Number of pups per litter in each group was maintained to avoid inflation due to sample size. Results were analyzed using one way repeated measures ANOVA. When day specific models reached the criterion of significance Post hoc tukey test was done. The significance level was set at p<0.001 to 0.05 as per requirement. The data were presented as Mean ± SEM.

## Figures and Tables

**Fig. 3.**
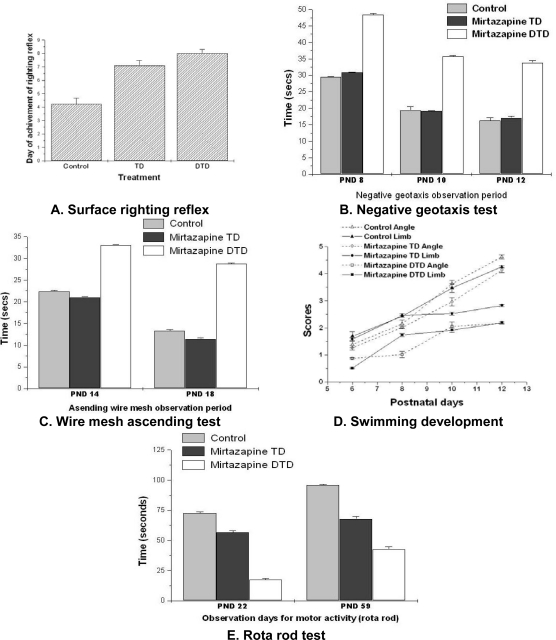
Effect of mirtazapine on motor development. A: DTD and TD exposed pup’s surface righting reflex was significantly (p<0.01) delayed than control. B: Swimming development of DTD exposed pups was significantly (p<0.01) poor than TD exposed pups and control. C: Negative geotaxis tests showed DTD exposed pups took significantly (p<0.01) more time than TD exposed pups and control. D: Ascending wire mesh test revealed DTD exposed pups took significantly (p<0.01) more time than TD exposed pups and control. E: Rota rod test showed DTD exposed pups spend significantly (p<0.05) less time on rod than TD exposed pups and control. Data analysis: one way ANNOVA followed by post hoc tukey.
